# GrainShape: A landmark-annotated image dataset of *japonica* rice grains for geometric morphometric analysis

**DOI:** 10.1016/j.dib.2025.111781

**Published:** 2025-06-11

**Authors:** Jiexiong Xu

**Affiliations:** College of Engineering, Academy for Advanced Interdisciplinary Studies, Nanjing Agricultural University, Nanjing 210095, China

**Keywords:** Rice grain morphology, Phenotyping, Landmarks, *Oryza sativa japonica* landraces

## Abstract

This open-access dataset comprises matched image and landmark files for 231 rice grains representing distinct *Oryza sativa japonica* landraces cultivated in China’s Lake Taihu region. Grains were grown in a single 2022 field season under uniform management. One mature, fully-filled kernel per accession was photographed dorsoventral side up with an Olympus TG-6 macro camera (4000 × 3000 px, 314 dpi, f/2.3, 1/13 s, +1 EV) under fixed laboratory lighting. Each frame includes a matte-green background, a 30 mm plastic ruler, and two printed fiducials, enabling pixel-to-millimeter scaling and consistent orientation. The repository is organized into four zip archives. grainimages.zip holds the 231 color JPEG photographs. landmarks.zip provides 231 plain-text tables listing the x,y coordinates of 30 homologous radial landmarks that capture length, width, curvature, tip geometry, shoulder position, awn insertion point, and subtle asymmetries. contours.zip contains 231 standalone PNG drawings showing a spline-interpolated outline reconstructed solely from each landmark set—no photographic background is included—facilitating rapid visual checks of digitization accuracy. scripts.zip contains all custom Python scripts for generating the landmark sets and reproducing the figures and tables presented in the paper. The dataset, available on Zenodo (DOI:10.5281/zenodo.15515366) under a CC BY 4.0 license, can be reused for landmark- or outline-based morphometric analysis, color and awn-length phenotyping, benchmarking of computer-vision pipelines, and instructional demonstrations of geometric morphometric workflows.

Specifications Table

**Table:** 

Subject	Biology
Specific subject area	Computer vision techniques for crop morphology and grain phenotyping .
Type of data	Image, scripts, and text file Raw, and analyzed.
Data collection	Digital grain images were acquired by trained researchers from Nanjing Agricultural University. One mature, filled grain was chosen from each of 231 japonica landrace accessions, thereby ensuring that every accession contributed a single, representative kernel. Grains were photographed with an Olympus TG-6 macro camera (4000 × 3000 px, 314 dpi, aperture f/2.3, exposure 1/13 s, +1 EV) under uniform, fixed lighting. Each grain was placed flat on a high-contrast matte-green background; a millimeter-scale calibration object and pre-printed reference points were included in every frame to enable subsequent metric scaling and landmark orientation. Images that were blurred, over-/under-exposed, or showed cracked, immature, or incomplete grains were excluded during quality control. All retained photographs were saved as color JPG files and later processed in ImageJ and custom Python scripts for landmark extraction and morphometric analysis*.*
Data source location	Data were collected at the experimental farm of Jiangsu Academy of Agricultural Sciences in Nanjing, Jiangsu Province, China (32.02° N, 118.46° E); all imaging and landmark digitization were carried out at the Nanjing Agricultural University, Nanjing (32.03° N, 118.84° E)*.*
Data accessibility	Repository name: ZENODO Data identification number: 10.5281/zenodo.15515366 Direct URL to data: https://zenodo.org/records/15515366
Related research article	none*.*

## 1 Value of the Data

• Comprehensive shape and awn morphometrics. The high-resolution images allow landmark- or outline-based extraction of grain length, width, curvature, tip sharpness, asymmetry, awn length, and other fine-scale geometric features across japonica varieties.
• Color-based phenotyping. Uniform lighting and color calibration enable quantitative analysis of grain and awn coloration (hue, saturation, brightness), supporting studies on pigment biochemistry, post-harvest quality, and consumer preference.
• Computer-vision and machine-learning development. The dataset provides a standard testbed for training and validating deep-learning or classical algorithms for automated seed phenotyping, variety classification, and defect detection.
• Benchmarking of image-analysis pipelines. Researchers can benchmark custom Python/R scripts or neural-network architectures on real, annotated grain images captured under controlled conditions.
• Educational and methodological use. The openly accessible images serve as ready-made examples for teaching geometric morphometrics, color analysis, and machine-vision techniques in plant science courses and workshops.

## 2 Background

Rice is the primary staple for more than half of the world’s population, yet breeding progress for consumer-preferred grain shape still relies on coarse length- and width-based indices [1]. Such one-dimensional measurements overlook tip sharpness, dorsal–ventral curvature, awn length, and subtle asymmetries that can affect milling yield, cooking quality, and market price [2]. Landmark-based geometric morphometrics now offers a rigorous framework for quantifying these complex outlines, and—when coupled with machine vision—promises scalable, objective phenotyping pipelines [3]. However, publicly available image sets that pair high-resolution grain photographs with verified landmark coordinates remain scarce, especially for Asian japonica landraces that constitute a valuable reservoir of shape diversity.

Several rice-grain image sets have been released in recent years, yet most focus on a handful of modern cultivars, offer low-resolution images, and present only de-hulled kernels. The Rice Image Dataset, for example, contains five commercial varieties photographed without hulls at 250 × 250 pixels and is intended solely for variety classification [4]. Çınar et al. provide two Turkish varieties, also de-hulled, for binary classification tasks [5]. Aruzz22.5K contributes 20 Bangladeshi cultivars (853 × 853 pixels), again hull-removed, for multi-class recognition [6]. Finally, A joint archive from BINA and BRRI offers images of 38 Bangladeshi varieties (640 × 480 pixels), likewise de-hulled and classification-oriented [7]. Geometric-morphometric work on rice has nevertheless advanced: Iwata et al. quantified grain outlines with elliptic Fourier descriptors [8], while Sakamoto et al. compared outline- and landmark-based methods for seed-shape characterization [9]. Open-source tools such as SHAPE and Momocs streamline descriptor extraction and statistical analysis [10]. Together, these studies highlight the power of high-precision shape analysis for exploring rice morphology and its variation.

**Fig. 1:**
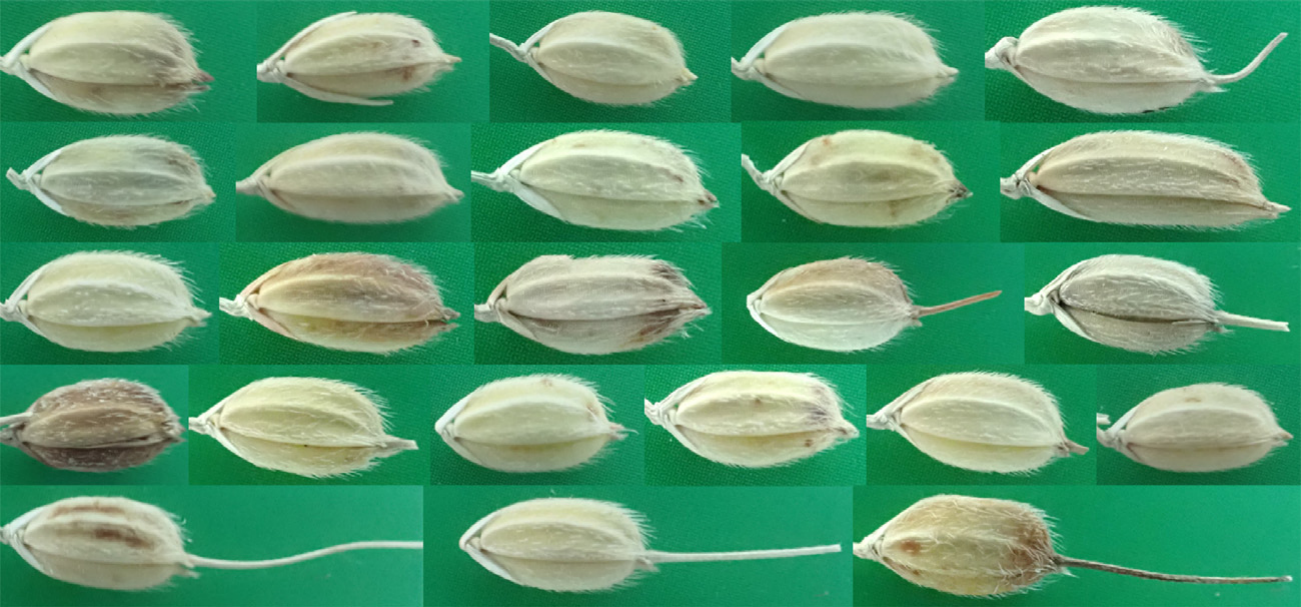
Composition of the GrainShape dataset. The dataset comprises 231 grain samples drawn from distinct Oryza sativa japonica landraces cultivated in the Lake Taihu region, China.

GrainShape addresses these limitations in three key ways. First, it spans a broad panel of 231 heritage japonica landraces—each withdrawn from commercial production but conserved in germplasm banks following many years of local selection for flavor, cultural preference, and micro‑environmental adaptation in the Lake Taihu region (Fig. 1). Second, every grain was photographed intact at 4000 × 3000 px under a controlled macro‑imaging setup that included a millimeter scale and color‑calibrated background, enabling the precise digitization of thirty homologous radial landmarks per image. Third, the inclusion of the hull makes it possible to quantify traits such as awn length, lemma–palea color contrasts, trichome density, and inner/outer bran‑boundary positions—measurements that are impossible in de‑hulled grains. By releasing raw photographs, landmark coordinate files, contour visualizations, and accompanying scripts, GrainShape offers a high-resolution benchmark for geometric-morphometric modelling, shape-based retrieval, transfer-learning workflows, and educational demonstrations—enabling users to customize and extend existing analysis pipelines.

## 3 Data Description

**Fig. 2:**
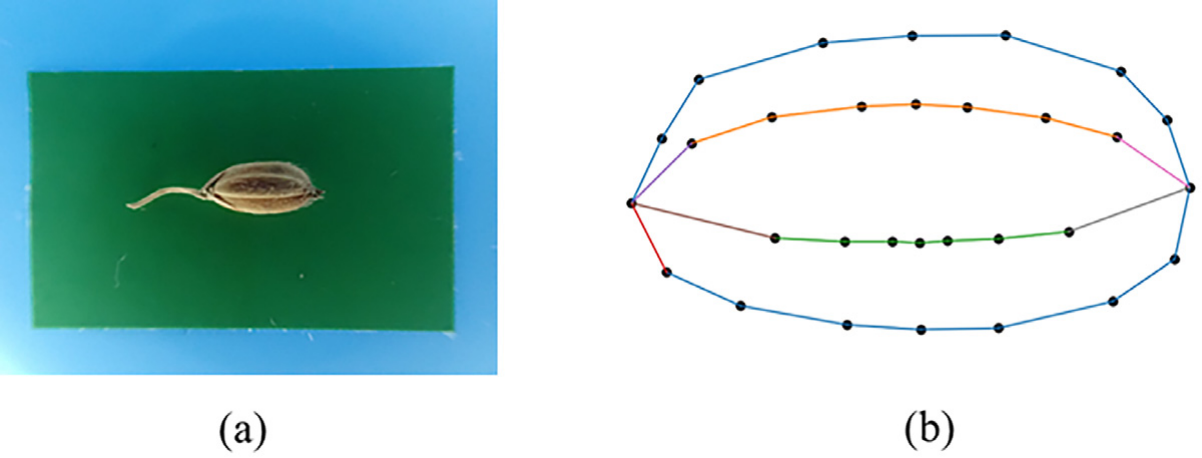
Illustration of raw grain image and landmark-derived contour. (a) Example JPEG photograph of a single Oryza sativa japonica grain on a matte-green background. (b) Spline-interpolated contour reconstructed solely from the 30 radial landmarks digitized on the grain outline; no photographic background is shown.

The dataset is distributed as four zipped archives that correspond to successive stages of data processing. grainimages.zip stores the raw phenotypic material: 231 color photographs of single rice grains collected from traditional japonica landraces grown around Lake Taihu. A green reference strip (30mm length) of known length is visible in every frame so that pixel distances can later be converted to millimeters and different images can be scaled to a common metric frame (Fig. 2a).

For each photograph there is a matching text file in landmarks.zip. These 231 plain-text files carry the same base names as their corresponding images and list the x–y pixel coordinates of the 30 homologous landmarks that trace the grain outline. Landmarks were placed according to the radial protocol described in Section Experimental Design, Materials and Methods, beginning at the distal tip and proceeding clockwise; the files therefore provide an ordered, machine-readable record of grain geometry that can be loaded directly into morphometric software.

The third archive, contours.zip, contains 231 PNG illustrations that overlay the landmark configuration and an interpolated spline on the original grain silhouette. These visualizations offer a quick, qualitative check of landmark placement accuracy and give users an immediate sense of the shape variation captured in the dataset. All folders follow identical naming conventions—e.g. T1.jpg, T1.txt, and T1.png—so that each grain’s image, coordinate set, and outline preview can be paired without ambiguity (Fig. 2b).

The fourth archive, scripts.zip, includes all custom Python scripts used to generate the landmark sets and reproduce the figures and tables presented in this paper (see “Script availability” below for full details).

## 4 Experimental Design, Materials and Methods

### 4.1 Data acquisition

The imaging campaign was coordinated solely by Nanjing Agricultural University. Rice plants representing 231 traditional japonica landraces were grown under uniform field conditions at the JAAS experimental farm, Nanjing, China (32.02 °N, 118.46 °E) during the 2022 season. At physiological maturity each landrace was gently air-dried, and a single, fully-filled grain—screened for intact hulls and typical size—was selected as that accession’s representative kernel.

Individual grains were photographed with an Olympus TG-6 digital macro camera mounted on a copy stand to ensure fixed geometry. Images were captured at 4000 × 3000 pixels (314 dpi) with the following settings: aperture f/2.3, exposure 1/13 s, +1 EV compensation, 5 mm focal length. Each grain was placed dorsoventral side up on a matte-green cardstock that maximized edge contrast. A millimeter-graduated plastic rule and two printed reference points were included in every frame to enable pixel-to-millimeter conversion and downstream alignment. Grains were oriented consistently (floral bract to the left, awn to the right) and the camera’s white balance was locked to laboratory lighting to maintain color fidelity. Photographs were saved on-board as 24-bit RGB JPEG files and immediately transferred to a secure workstation for inspection.

### 4.2 Landmark annotation

**Fig. 3:**
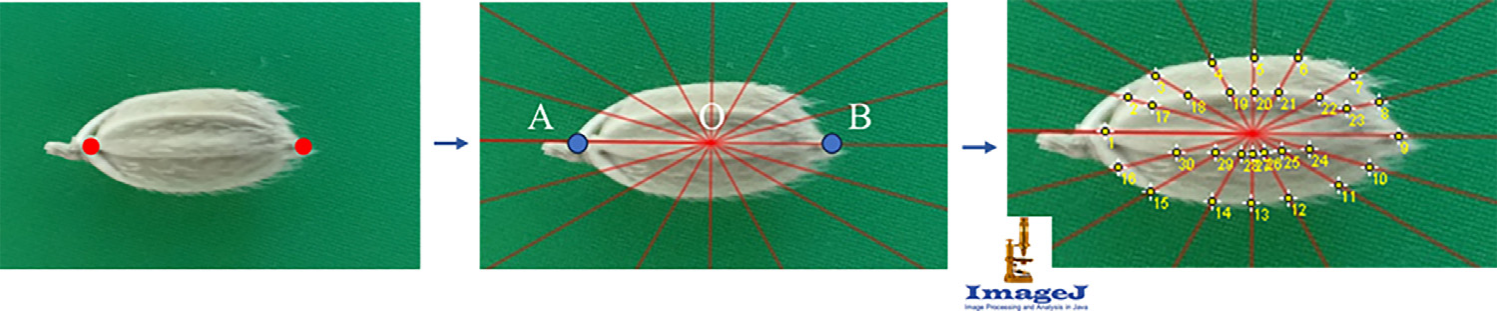
Landmark annotation workflow

Raw photographs were screened for focus, exposure and kernel integrity; any image showing blur, shadows or cracked grains was discarded. Accepted files were renamed with a stable key (e.g., T1.jpg) that links the image to its accession metadata. Using ImageJ, operators digitized 30 homologous landmarks per grain: the left and right apices defined a baseline (points *A* and *B*) and 14 predefined radial lines (15°–345°) emanating from the midpoint (*O*) intersected the grain outline to yield the remaining points. Landmark coordinates were exported as plain-text tables (T1.txt) (Fig. 3).

For visual quality control a spline contour was interpolated through each 30-point configuration; the resulting PNG overlays (T1.png) allow rapid assessment of landmark placement accuracy. Finally, the workflow packaged the vetted JPGs, landmark text files and contour overlays into three archives—grainimages.zip, landmarks.zip and contours.zip.

### 4.3 Landmark standardization

To remove between-image differences in position and orientation, the raw landmark sets were subjected to a rigid (translation + rotation) alignment. For each specimen the midpoint *O* of the baseline *AB* (apex-to-apex segment defined above) was taken as the temporary origin. Every landmark coordinate was then translated so that *O* coincided with the origin, thereby eliminating positional differences among images. Next, the angle *θ* between the segment *AB* and the positive x-axis was calculated as:

$$\theta =\;\text{arctan}\left(\frac{y_{B}-y_{A}}{x_{B}-x_{A}}\right)$$

All translated coordinates (x,y) were rotated by –*θ* using the 2 × 2 matrix:

$$R=\left[\begin{array}{cc} \cos\theta & \sin\theta \\ -\sin\theta & \cos\theta \end{array}\right]$$

This operation aligns the longitudinal axis horizontally while retaining the original metric scale. The procedure is implemented in standardize_landmarks.py (see scripts/); the resulting aligned coordinates for all 231 grains are provided in std_landmarks.txt and were used for further analyses.

### 4.4 Data quality analysis

**Fig. 4:**
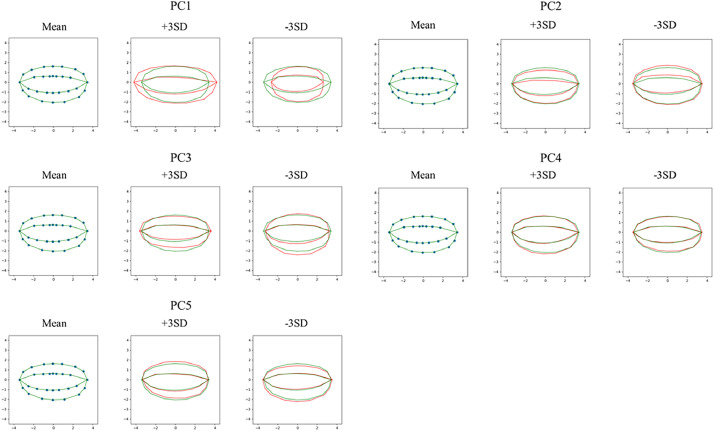
Grain morphology analysis for PC1–PC5. Mean shape and variation along each principal component (PC1–PC5). Each panel represents one principal component. For each principal component, the plot on the left shows the mean grain shape (represented by blue landmarks), while the plots on the right illustrate the grain shapes at +3 standard deviations (SD, red) and -3 standard deviations (green) along the corresponding principal component. These visualizations highlight the shape variations captured by each PC, revealing the impact of each component on grain morphology.

To evaluate how well the 30 landmark points capture grain shape variation, a principal component analysis (PCA) was conducted on the landmark coordinate matrices. The first five principal components together account for 87.71 % of the total variance—PC1 (41.48 %), PC2 (21.30 %), PC3 (11.67 %), PC4 (7.03 %), and PC5 (6.23 %)—demonstrating that the chosen landmarks cover both major and finer-scale outline features (Fig. 4).

Following the centroid‑distance signature concept widely used in computer vision and morphometrics, we measured straight‑line radii (dist1–dist8) at fixed angles from the grain midpoint. Although these values are not formal differential curvatures, shorter radii indicate locally flatter bending of the hull, whereas longer radii correspond to tighter regions. Such centroid‑to‑outline distances have long been employed as practical curvature surrogates in shape studies. This radial‑distance strategy follows the centroid‑signature approaches of Belongie et al. [11] and Ling & Jacobs [12], and has been applied to rice grains in the SmartGrain pipeline [13].

**Fig. 5:**
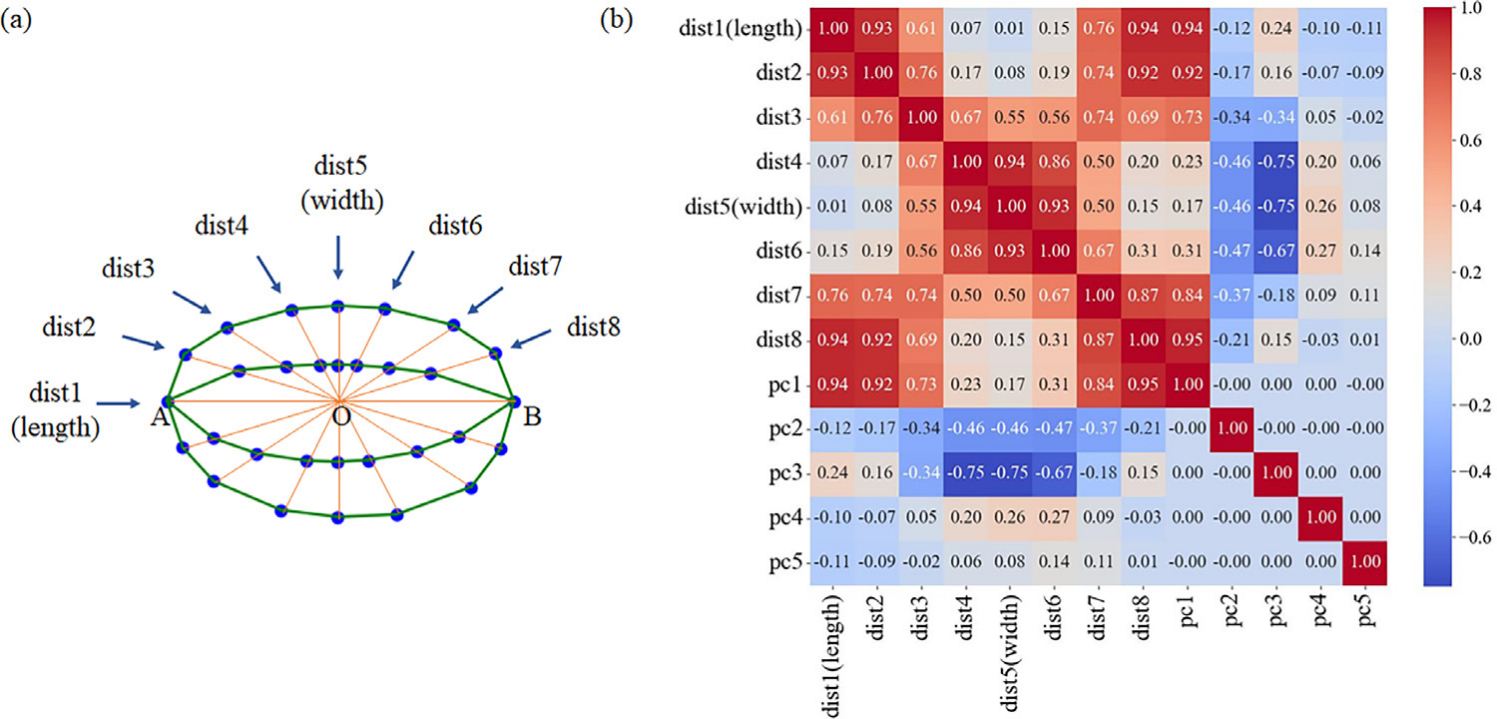
Correlation analysis between grain measurement distances and principal components (PC1–PC5). (a) Schematic diagram showing the radial measurement distances from the center of the rice grain. “dist1” and “dist5” represent the standard length and width measures, respectively. The remaining six radial lines, labelled “dist2” through “dist8,” represent the grain's curvature parameters, capturing straight-line distances at various angles from the center. (b) Heatmap of the correlation analysis between the grain measurement distances (dist1 to dist8) and the first five principal components (PC1–PC5).

Each of the eight distances is anchored on the longitudinal axis AB that joins the apex and the basal hilum. Taking the midpoint O of AB as the origin, we project eight fixed rays into the outline. Dist1 is measured along AB (0°) and represents grain length, whereas dist5 is drawn at 90° and represents grain width. Three additional dorsal rays are cast at +15°, +30°, and +60° (dist2, 3, 4) and their ventral counterparts at –15°, –30°, and –60° (dist8, 7, 6). Because the oblique rays intersect progressively steeper regions of the grain, a longer centroid‑to‑outline radius corresponds to a more sharply curved segment, whereas a shorter radius indicates a flatter portion of the grain. Accordingly, in subsequent analyses we refer to dist2–dist4 and dist6–dist8 collectively as curvature parameters, whereas dist1 and dist5 are treated as the length and width measures (Fig. 5a).

**Fig. 6:**
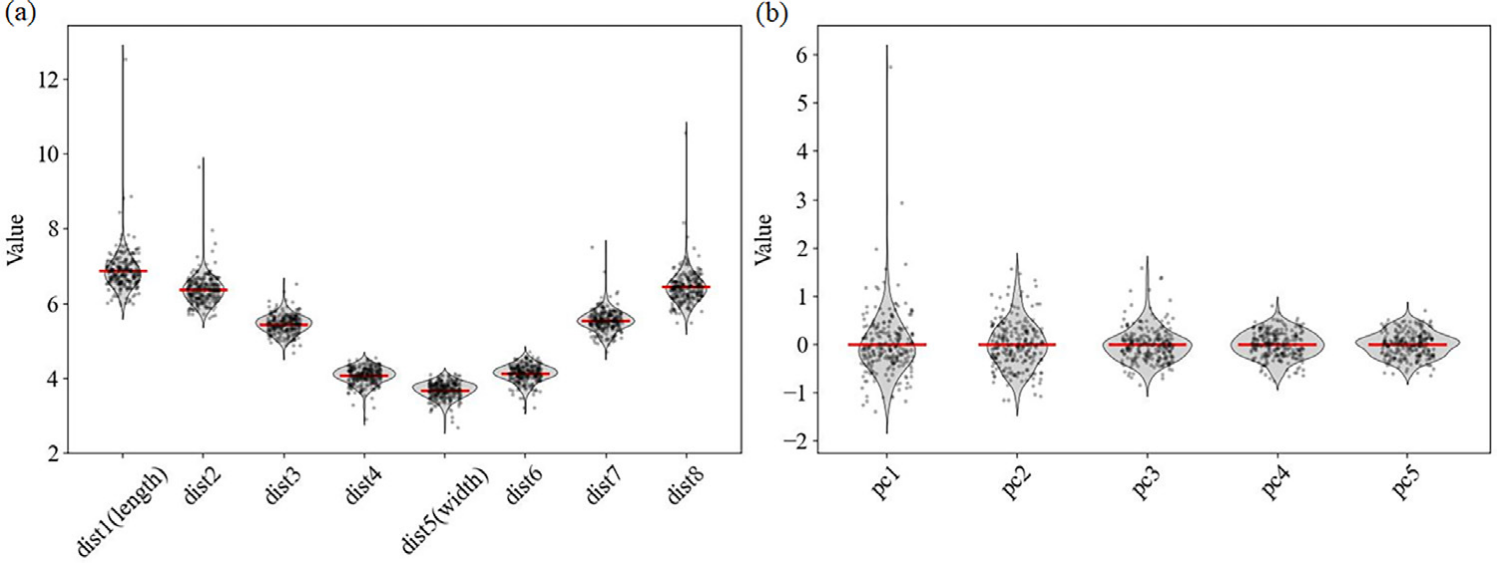
Grain-shape violin plots. (a) Eight linear distances (dist1–dist8) and (b) the first five principal component scores (PC1–PC5) for all 231 grains. Each grey violin represents the kernel density distribution of a trait; its width reflects the local probability density. Black contours outline the violins, jittered black dots show individual grain values, and the red horizontal line indicates the trait mean. Axes use independent vertical scales.

Violin plots (Fig. 6) and summary statistics (Table. S1) were added for the eight centroid‑to‑outline distances (dist1–dist8) and for PC1–PC5 to characterize overall variation. Moderate dispersion was observed among the linear measures: the coefficient of variation for grain length was 8.30 % (range 5.96–12.55 mm) and for grain width 6.27 %, underscoring differences among landraces. The largest variation among the principal components appeared in PC1 (SD = 0.68; range –1.39 to 5.75), with PC2 showing a smaller yet notable spread (SD = 0.48; range –1.14 to 1.57). These metrics confirm pronounced inter‑sample diversity across both traditional distance‑based traits and multivariate shape components.

Next, Pearson correlation coefficients were calculated between each PC score and the eight linear measurements (grain length, grain width, and six curvature parameters). PC1 showed strong correlations with length-related traits (*r* = 0.73 to 0.95) but weak associations with width (*r* = 0.17 to 0.31), indicating that it primarily captures variation along the longitudinal axis. This pattern aligns with known developmental mechanisms regulating grain elongation, where differential cell expansion in early caryopsis development drives longitudinal growth without proportional changes in width [14,15]. PC2 shows moderate correlations with overall grain width (*r* = –0.46 to –0.47), but inspection of Fig. 4 reveals that its deformation vectors predominantly widen the palea flank, indicating transverse expansion on that side. Conversely, PC3—although more strongly correlated with width (*r* = –0.67 to –0.75)—drives widening on the lemma flank, pointing to asymmetrical tissue growth that shifts lateral mass toward the lemma. Taken together, PC2 and PC3 therefore capture transverse growth patterns consistent with previously reported, coordinated cell-proliferation pathways [[16], [17], [18]]. PC4 displayed moderate correlations with dorsoventral curvature parameters (*r* = 0.20–0.27), consistent with differential endosperm deposition during late grain filling, which mechanically biases hull curvature toward the dorsal side[19]. PC5 showed minimal correlations with linear metrics (|*r*|_max_ = 0.14) but revealed subtle symmetry shifts in deformation grids. This phenomenon may reflect developmental perturbations during grain formation, as similar lateral asymmetries have been documented in studies of rice grain morphology[20]. Collectively, these relationships (Fig. 5b) demonstrate that the landmark configuration captures biologically meaningful size, and curvature components of japonica grain morphology.

### 4.5 Script availability

All Python scripts required to reproduce the measurements, figures and supplementary tables are provided in the scripts/ directory of the Zenodo record. A concise workflow is outlined below; only the root path in each script needs to be adjusted to the user’s local environment.

Reference‑line generation and landmarking: select_ab_generate_reference_line.py reads the images in grainimages/, prompts the user to click the apex and basal poles, and writes the AB axis together with the derived radial reference lines. This workflow first generates grain images overlaid with the reference lines; landmarks—defined by the intersections of these rays with the grain outline and the inner/outer bran boundaries (Fig. 3)—are then annotated on these images using ImageJ. The resulting coordinates used in the article are available in landmarks/.

Pixel‑to‑millimeter scaling: measure_label_pixel_length.py measures the label bar in each reference‑line image and records pixel length. The compiled values and the converted physical‑scale landmarks are stored in …/landmarks_scaled_to_mm.xlsx and …/landmarks.txt.

Rigid alignment: The 231 landmark sets are translated and rotated into a common coordinate system with standardize_landmarks.py; the aligned matrix used in the analyses is provided as …/std_landmarks.txt.

Metric extraction: compute_dist1_to_dist8.py and compute_pc1_to_pc5.py calculate grain length, width, the six curvature parameters (dist1–dist8), and the first five principal components. The output table (…/dist1_to_dist8_pc1_to_pc5_values.xlsx) reproduces the values reported in this article.

Figure generation: generate_grain_contours.py draws the contour visualizations saved in contours/. generate_pca_plots.py produces the PCA deformation grids (Fig. 4). generate_correlation_heatmap.py renders the PC–distance correlation heat‑map (Fig. 5b). generate_violin_plots.py outputs the grain‑shape violin plots (Fig. 6).

Shape‑based clustering: landmark_shape_clustering.py reads the aligned landmark matrix (…/std_landmarks.txt), treats each 60‑dimensional sample as a shape vector, and performs both KMeans and hierarchical (Ward) clustering. It outputs cluster labels for each sample and generates a dendrogram to guide selection of cluster number based on shape similarity. This script is provided as a demo of landmark-based clustering; users can adapt it to suit their own research needs.

Users may either supply their own landmark files or work directly with the processed datasets in scripts/processed data/. All scripts are fully commented to facilitate modification and extension.

## Limitations

This dataset includes only two-dimensional images of rice grains from one side, so information about grain thickness or three-dimensional shape is not captured. The imaging setup was optimized for uniformity, but slight variations in lighting or focus may remain. Also, only the morphological data (images) are provided; no genetic, environmental, or agronomic metadata accompany the images. Therefore, analyses using this dataset should be limited to shape phenotyping and should not attempt to infer genetic relationships without additional data.

## Ethics Statement

The authors have read and follow the ethical requirements for publication in Data in Brief. The current work does not involve human subjects, animal experiments, or any data collected from social media platforms.

## CRediT Author Statement

**Jiexiong Xu:** Conceptualization; Data curation; Methodology; Formal analysis; Investigation; Writing – original draft; Writing – review & editing.

## Data Availability

ZENODOGrainShape: a landmark-annotated image dataset of japonica rice grains for geometric morphometric analysis (Original data)

## Appendix Supplementary materials

Image, application 1
