# Supplementary material for: GrainShape: A landmark-annotated image dataset of japonica rice grains for geometric morphometric analysis
Source: Data Brief. 2025 Jun 11;61:111781. doi: 10.1016/j.dib.2025.111781 (PMC12221751; doi:10.1016/j.dib.2025.111781)
Supplement: Supplementary file 1 [file mmc1.docx]

**Table S1.** Descriptive statistics of grain phenotypic traits from image analysis in the rice landrace panel.

| Trait | Mean | Median | Std | 1st Qu | 3rd Qu | Min | Max |
| --- | --- | --- | --- | --- | --- | --- | --- |
| dist1(length) | 6.87 | 6.82 | 0.57 | 6.59 | 7.07 | 5.96 | 12.55 |
| dist2 | 6.37 | 6.36 | 0.4 | 6.14 | 6.55 | 5.61 | 9.65 |
| dist3 | 5.44 | 5.46 | 0.26 | 5.29 | 5.6 | 4.66 | 6.52 |
| dist4 | 4.06 | 4.09 | 0.24 | 3.94 | 4.22 | 2.9 | 4.54 |
| dist5(width) | 3.67 | 3.7 | 0.23 | 3.54 | 3.83 | 2.67 | 4.11 |
| dist6 | 4.12 | 4.14 | 0.24 | 4 | 4.28 | 3.2 | 4.7 |
| dist7 | 5.54 | 5.53 | 0.3 | 5.38 | 5.7 | 4.69 | 7.5 |
| dist8 | 6.43 | 6.41 | 0.45 | 6.19 | 6.6 | 5.47 | 10.56 |
| pc1 | 0 | -0.09 | 0.68 | -0.35 | 0.25 | -1.39 | 5.75 |
| pc2 | 0 | -0.03 | 0.48 | -0.31 | 0.29 | -1.14 | 1.57 |
| pc3 | 0 | -0.02 | 0.36 | -0.25 | 0.15 | -0.83 | 1.59 |
| pc4 | 0 | 0 | 0.28 | -0.19 | 0.2 | -0.75 | 0.81 |
| pc5 | 0 | 0.01 | 0.26 | -0.21 | 0.16 | -0.65 | 0.71 |
